# The Ror-Family Receptors in Development, Tissue Regeneration and Age-Related Disease

**DOI:** 10.3389/fcell.2022.891763

**Published:** 2022-04-13

**Authors:** Mitsuharu Endo, Koki Kamizaki, Yasuhiro Minami

**Affiliations:** Division of Cell Physiology, Department of Physiology and Cell Biology, Graduate School of Medicine, Kobe University, Kobe, Japan

**Keywords:** non-canonical wnt signaling, cell polarity, migration, proliferation, stem/progenitor cells, cellular senescence, inflammation, cancers

## Abstract

The Ror-family proteins, Ror1 and Ror2, act as receptors or co-receptors for Wnt5a and its related Wnt proteins to activate non-canonical Wnt signaling. Ror1 and/or Ror2-mediated signaling plays essential roles in regulating cell polarity, migration, proliferation and differentiation during developmental morphogenesis, tissue-/organo-genesis and regeneration of adult tissues following injury. Ror1 and Ror2 are expressed abundantly in developing tissues in an overlapping, yet distinct manner, and their expression in adult tissues is restricted to specific cell types such as tissue stem/progenitor cells. Expression levels of *Ror1* and/or *Ror2* in the adult tissues are increased following injury, thereby promoting regeneration or repair of these injured tissues. On the other hand, disruption of Wnt5a-Ror2 signaling is implicated in senescence of tissue stem/progenitor cells that is related to the impaired regeneration capacity of aged tissues. In fact, Ror1 and Ror2 are implicated in age-related diseases, including tissue fibrosis, atherosclerosis (or arteriosclerosis), neurodegenerative diseases, and cancers. In these diseases, enhanced and/or sustained (chronic) expression of Ror1 and/or Ror2 is observed, and they might contribute to the progression of these diseases through Wnt5a-dependent and -independent manners. In this article, we overview recent advances in our understanding of the roles of Ror1 and Ror2-mediated signaling in the development, tissue regeneration and age-related diseases, and discuss their potential to be therapeutic targets for chronic inflammatory diseases and cancers.

## Introduction

In the developmental process, various cell behaviors, including cell polarization, migration, proliferation, and differentiation, are strictly regulated based on genetic programs, and thereby establishing proper morphogenesis and tissue-/organo-genesis. The regulation of these cell behaviors is also essential for proper tissue repair or regeneration following injury even in the adult tissues. For example, tissue stem/progenitor cells initiate their proliferation and differentiation in response to local environmental changes including inflammation caused by injury (Kizil et al., 2015; Xiao et al., 2020). The signaling mechanisms involved in the regulation of the developmental processes and responses following injury should be activated or inhibited at the appropriate strength and timing. Therefore, their aberrant activation under the pathological conditions such as chronic inflammation with aging might contribute to the progression of various diseases.

The Ror-family proteins, Ror1 and Ror2 act as receptors or co-receptors for non-canonical Wnt ligands such as Wnt5a, thereby regulating cell polarity, migration, proliferation, and differentiation that are required for proper developmental morphogenesis and tissue-/organo-genesis (Minami et al., 2010; Endo and Minami, 2018). *Ror1* and *Ror2* are expressed selectively in some undifferentiated cells including stem/progenitor cells, implying that in addition to the amount of their ligands, the gene regulation of the Ror-family receptors is important for their actions. In this review, we first overview the functions of the Ror-family receptors in regulating developmental processes, and then introduce how expression of *Ror1* and/or *Ror2* is regulated in certain types of cells, including tissue-resident stem/progenitor cells, in adult tissues during tissue repair or regeneration following injury. We also discuss the possible involvement of aberrant signaling mediated by the Ror-family receptors in the stem/progenitor cell aging and the age-related diseases including chronic inflammatory diseases and cancers.

## Functional Domains of the Ror-Family Proteins in Regulating Non-canonical Wnt Signaling

### Extracellular Domains

**FIGURE 1:**
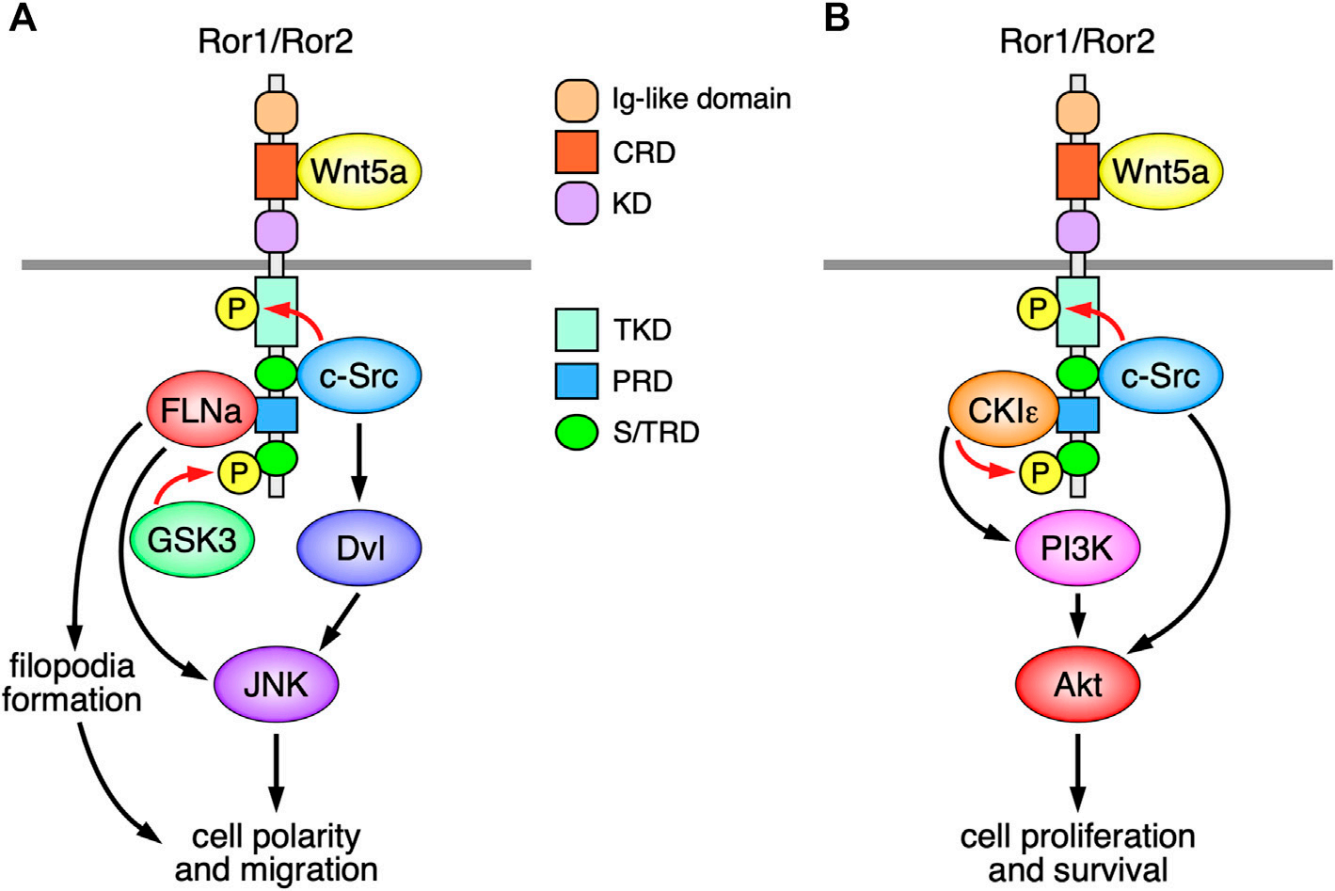
Schematic representation of the Ror-family receptors (Ror1/Ror2)-mediated signaling, implicated in the regulation of Wnt5a-induced cell polarity, migration, proliferation, and survival. **(A)** The Ror-family receptors induce filopodia formation and JNK activation via the interaction with FLNa, leading to the regulation of cell polarity and migration. Wnt5a-Ror signaling can also induce activation of c-Src and Dvl, leading to the activation of JNK. c-Src and GSK3, respectively, phosphorylate the Ror-family receptors on their tyrosine and serine/threonine residues, both of which are required for Wnt5a-induced cell polarity and migration. **(B)** Wnt5a-Ror signaling can activate PI3K-Akt pathway presumably via CKIε associated with Ror1/Ror2, which in turn promotes cell proliferation and survival. c-Src, activated by Wnt5a-Ror signaling, can also induce activation of Akt, thereby contributing to cell proliferation and survival.

In vertebrates, the Ror-family receptors consist of two structurally related proteins, Ror1 and Ror2. Both Ror1 and Ror2 (hereinafter, referred to as Ror1/Ror2) are type I transmembrane proteins, and their extracellular regions are composed of an immunoglobulin (Ig)-like domain, followed by a frizzled-like cysteine-rich domain (CRD), and then a kringle domain (KD) (Figure 1). The CRDs of Ror1/Ror2 can interact with Wnt5a (Oishi et al., 2003; Mikels and Nusse, 2006; Weissenböck et al., 2019), one of the most extensively studied non-canonical Wnt proteins. A growing body of evidence shows that Ror1/Ror2 act as receptors or co-receptors for various Wnt proteins, including Wnt5a, Wnt5b, Wnt9a, and Wnt11, to regulate various cellular responses (Oishi et al., 2003; Mikels and Nusse, 2006; Fukuda et al., 2008; Morioka et al., 2009; Paganoni et al., 2010; Sato et al., 2010; Endo et al., 2012; Ho et al., 2012; Bai et al., 2014; Weissenböck et al., 2019; Menck et al., 2021). The KD of Ror1 can interact with that of Ror2 to form Ror1/Ror2 heterooligomers that are required for Wnt5a-induced signaling at least in some cellular contexts (Yu et al., 2016). Although the function of the Ig-like domain in the Ror-family receptors remains elusive, *Ror1* mutant mice lacking the Ig-like domain exhibit abnormal development of the kidneys when crossed with *Ror2* deficient mice or *Wnt5a* heterozygous mutant mice (Qi et al., 2016), suggesting that Ror1/Ror2 have redundant functions in regulating Wnt5a-induced signaling.

### Cytoplasmic Domains

The cytoplasmic regions of the Ror-family receptors contain a putative conserved tyrosine kinase domain (TKD), followed by a serine/threonine-rich domain (S/TRD1), a proline-rich domain (PRD), and another serine/threonine-rich domain (S/TRD2) (Figure 1). Recent studies demonstrate that the TKDs of Ror1/Ror2 act as pseudokinase domains that are incapable of binding to ATP by itself (Sheetz et al., 2020), although they can mediate Wnt5a-induced signaling and cellular responses (Enomoto et al., 2009; Sheetz et al., 2020). It has been shown that the non-receptor tyrosine kinase c-Src can phosphorylate the tyrosine residues within the activation loops in the TKDs of Ror1/Ror2 (Akbarzadeh et al., 2008; Gentile et al., 2014), and that the substitutions of these tyrosine residues with phenylalanine result in the impairment of their several functions (Mikels et al., 2009; Gentile et al., 2014; Sheetz et al., 2020). However, further studies will be required to determine the roles of the tyrosine phosphorylation within the TKDs of Ror1/Ror2 in regulating various cellular behaviors.

The S/TRD1 of Ror2 is required for recruitment and activation of c-Src following Wnt5a-stimulation (Akbarzadeh et al., 2008; Lai et al., 2012). Accumulating evidence demonstrates that c-Src plays essential roles in regulating Ror1/Ror2-mediated cell migration and proliferation (Enomoto et al., 2009; Gentile et al., 2014; Shojima et al., 2015). Ror2 can also bind to other signaling molecules, including filamin A (FLNa) and casein kinase I epsilon (CKIε), via the PRD (Kani et al., 2004; Nishita et al., 2006). Ror2 mediates filopodia formation and c-Jun N-terminal kinase (JNK) activation via the interaction with FLNa, thereby regulating cell polarity and migration (Nishita et al., 2006; Nomachi et al., 2008) (Figure 1A). Interestingly, c-Src has also been implicated in the activation of Dishevelled (Dvl) and c-Jun N-terminal kinase, downstream of Wnt5a-Ror2 signaling (Yamagata et al., 2012) (Figure 1A). Like Ror2, Ror1 might exhibit the same or similar function in regulating polarized cell migration presumably through filopodia formation (Kaucka et al., 2011). The serine/threonine residues within the C-terminal portion of Ror1/Ror2 can be phosphorylated via glycogen synthase kinase 3 (GSK3) and CKIε, which might be required for the activation of non-canonical Wnt signaling (Kani et al., 2004; Yamamoto et al., 2007; Grumolato et al., 2010; Konopelski Snavely et al., 2021). Indeed, it has been shown that GSK3-mediated phosphorylation of Serine 864 within the STD2 of Ror2 is required for Wnt5a-induced cell migration (Grumolato et al., 2010) (Figure 1A), and that Wnt5a-Ror signaling can also activate phosphoinositide three kinase (PI3K)-Akt pathway presumably via CKIε associated with Ror1/Ror2, which in turn promotes cell proliferation and survival (Zhang et al., 2012) (Figure 1B).

## The Ror-Family Receptors in Development

### Embryonic Morphogenesis

Wnt signaling can largely be classified into canonical (*β*-catenin-dependent) and non-canonical (*β*-catenin-independent) pathways. Wnt5a can activate the non-canonical Wnt/planar cell polarity (PCP) pathway, in which Dvl activates the Rho-family of small GTPases, including Rho, Rac, and Cdc42, and their downstream effectors, Rho-associated protein kinase and JNK, leading to reorganization of the actin cytoskeleton, and thereby regulating polarized cell morphology and migration. In vertebrates, Wnt/PCP pathway regulates polarized cell movements during convergent extension (CE), by which tissues undergo narrowing along one axis and concomitant extension along another axis. Indeed, Wnt5a-Ror2 signaling can regulate CE movements during gastrulation and neurulation in *Xenopus* embryos (Hikasa et al., 2002; Oishi et al., 2003; Schambony and Wedlich, 2007; Martinez et al., 2015). In zebrafish, *Wnt11*, but not *Wnt5a*, is expressed highly at gastrula-stage embryos and Wnt11-Ror2 signaling is implicated in regulating CE movements during gastrulation (Bai et al., 2014).

In mammals, Wnt/PCP pathway plays essential roles in regulating collective and directed cell movements involved in various developmental processes, including neural tube closure, neural crest migration, the anterior to posterior axis elongation, inner ear hair cell alignment, and heart morphogenesis (Kibar et al., 2001; Curtin et al., 2003; Montcouquiol et al., 2003; Wang J. et al., 2006; Wang Y. et al., 2006; Ybot-Gonzalez et al., 2007; Muñoz-Soriano et al., 2012). Indeed, *Ror2* ^−/−^ and *Wnt5a* ^−/-^ mouse embryos exhibit dwarfism, short limbs and tail, facial anomalies, and ventricular septal defect (Yamaguchi et al., 1999; DeChiara et al., 2000; Takeuchi et al., 2000; Oishi et al., 2003; Schwabe et al., 2004; He et al., 2008), reminiscent of features observed in patients with Robinow syndrome that is caused by loss-of-function mutations in *Ror2* or *Wnt5a* genes (Atalay et al., 1993; Afzal et al., 2000; van Bokhoven et al., 2000; Person et al., 2010). These mutant mice also exhibit disrupted alignment and orientation of inner ear hair cells (Qian et al., 2007; Yamamoto et al., 2008), and disrupted morphogenesis of the kidney, lung, trachea, esophagus, and midgut (Yamada et al., 2010; Kishimoto et al., 2018). Although the phenotypes of *Ror2* ^−/−^ mouse embryos are somewhat milder than those of *Wnt5a* ^−/-^ mouse embryos, severer morphological phenotypes are observed in *Ror1*/*Ror2* double mutant mice (Nomi et al., 2001; Ho et al., 2012), indicating that pleiotropic, yet redundant functions of *Ror1*/*Ror2* during developmental morphogenesis.

### Tissue- and Organo-Genesis

During mouse development, Ror1/Ror2 are expressed in various tissues and organs, including the lung, kidney, tooth, and the skeletal and nervous systems (Oishi et al., 1999; Takeuchi et al., 2000; Al-Shawi et al., 2001; Matsuda et al., 2001), and play crucial roles in their establishment by regulating cell proliferation and differentiation as well as cell polarity and migration (Schwabe et al., 2004; Lin et al., 2011; Maeda et al., 2012; Nishita et al., 2014; Zhang K. et al., 2020; Ma et al., 2021).

### Nervous System

In the nervous system of mouse embryos, *Ror1*/*Ror2* are expressed in neural stem/progenitor cells (NPCs) within the neocortices (Oishi et al., 1999; Al-Shawi et al., 2001; Endo et al., 2012). At early embryonic stages, NPCs divide actively to maintain the NPC pool and thereby generating a large number of neurons via intermediate progenitor cells (IPCs), neuron-producing transient amplifying cells. Suppressed expression of *Ror1* and/or *Ror2* in NPCs results in reduced proportion of proliferating NPCs, IPCs, and their progeny neurons, probably due to increased cell cycle exit (Endo et al., 2012). In regulating the cell-cycle, Ror2-signaling can promote cell-cycle transition from the G1 to S phase by activating E2F1-mediated transcription (Endo et al., 2020). Expression levels of *Ror1*/*Ror2* in NPCs are decreased gradually during neocortical development (Endo et al., 2017), suggesting that the relative activity of Wnt5a-Ror1/Ror2 signaling in NPCs might play a role in determining durations of the neocortical neurogenesis.

### Skeletal System

*Ror2* ^−/−^ mice exhibit short limbs with mesomelic dysplasia characterized by significant or complete loss of the distal long bones in the forelimbs (the radius and ulna) and hindlimbs (the tibia and fibula) (DeChiara et al., 2000; Takeuchi et al., 2000). Although *Ror1* mutant mice do not show any apparent skeletal abnormalities, *Ror1/Ror2* double mutant mice exhibit a drastic enhancement of the skeletal phenotypes observed in the *Ror2* ^−/−^ mice and additional phenotypes (Nomi et al., 2001), indicating that *Ror1* and *Ror2* interact genetically in regulating the developmental bone growth. Longitudinal growth of the bones is achieved by endochondral ossification, where a pre-formed cartilage template is replaced by newly formed bone. During this process, chondrocytes, which are derived from undifferentiated mesenchymal cells, proliferate and differentiate into hypertrophic chondrocytes within the cartilage template, subsequently instruct the differentiation of adjacent perichondrial cells into osteoblasts. *Ror2* is expressed selectively in the resting and proliferating chondrocytes but not hypertrophic chondrocytes (DeChiara et al., 2000), and loss of *Ror2* in limb bud mesenchyme leads to a decrease in chondrocyte differentiation and impaired ossification in the developing cartilages (Schwabe et al., 2004; Lei et al., 2020).

### Tooth

During embryonic stages of mouse tooth development, *Ror2* is expressed in both the dental epithelium and mesenchyme, while *Wnt5a* is expressed only in the mesenchyme within the dental papilla adjacent to the dental epithelium (Lin et al., 2011). Teeth of *Ror2* ^−/−^ and *Wnt5a* ^−/-^ mice at P0 exhibit retarded growth with a delayed odontoblast differentiation (Lin et al., 2011). After the development of the dental crown, tooth root formation occurs extensively during the postnatal period in mice. Expression of Ror2 is widely distributed in the dental epithelium and mesenchyme at P0, but gradually become more prominent apically in the root-forming region during the root development of neonatal mice (Ma et al., 2021). Conditional deletion of *Ror2* in the dental mesenchyme resulted in shortened roots without obvious abnormality in the crown patterning (Ma et al., 2021), suggesting that Ror2 expressed in the dental mesenchyme and epithelium contributes to the formation of the tooth root and dental crown, respectively. During root development, Ror2 signaling is required for cell proliferation of the dental mesenchyme and differentiation of odontoblasts (Ma et al., 2021).

## The Ror-Family Receptors in Tissue Regeneration and Repair

### Regeneration of Muscle

Although overall expression levels of the Ror-family receptors in adult tissues are reduced compared to developing tissues, expression of *Ror1* can be detected in satellite cells (SCs), one of the skeletal muscle-specific tissue stem cells, within the skeletal muscles of the adult mice (Kamizaki et al., 2017). It is well known that SCs play a crucial role in regulating the regeneration of skeletal muscles after injury (Mauro, 1961; Chargé and Rudnicki, 2004). Although SCs are in quiescent state under physiological conditions, they are activated and initiate their proliferation following skeletal muscle injury, then differentiate and fuse with each other to produce new myofibers, thereby promoting skeletal muscle regeneration (Seale et al., 2000). Importantly, expression level of *Ror1* in SCs is further increased upon skeletal muscle injury (Kamizaki et al., 2017). Tumor necrosis factor-α and interleukin-1β (IL-1β), inflammatory cytokines produced from inflammatory cells, including neutrophils and macrophages infiltrated into the injured muscles, can induce the expression of *Ror1* in SCs through the activation of NF-κB pathway. Further studies in SCs-specific *Ror1* conditional knockout-mice have revealed that increased expression of *Ror1* in SCs is required for their proliferation following injury and subsequent regeneration of skeletal muscles (Kamizaki et al., 2017).

### Repair in Nervous System

The adult mammalian brains have a limited capacity to regenerate spontaneously following injury or diseases. Although NPCs exist in some parts even in the adult mammalian brains, there are no stem/progenitor cells involved in generating new neurons to compensate damaged neurons at least within the injured parenchyma. It has been shown that *Ror2* expression is increased in the brains following traumatic injury (Endo et al., 2017). Interestingly, increased expression of Ror2 is observed in reactive astrocytes, surrounding the injured sites, that express Nestin, a marker of NPCs. Astrocytes are the most abundant glial cells in the brain and contribute to brain homeostasis. Under pathological conditions, astrocytes change to a state called reactive astrocytes that exhibit various specific properties, thereby affecting the brain functions. In the injured brains, reactive astrocytes acquire stem cell-like properties and start to proliferate (Götz et al., 2015). It has been reported that proliferative reactive astrocytes exhibit anti-inflammatory and tissue-repair promoting functions (Sofroniew, 2015; Frik et al., 2018; Williamson et al., 2021). In astrocyte-specific *Ror2* conditional knockout-mice, number of proliferating reactive astrocytes is decreased (Endo et al., 2017), indicating that induced expression of *Ror2* in reactive astrocytes plays an important role in promoting repair of the injured brains.

Cell-cycle entry of quiescent astrocytes is triggered by growth factors, including basic fibroblast growth factor (bFGF), where expression of *Ror2* can be induced during the transition from G0/G1 to S-phase (Endo et al., 2017). It has been shown that *Ror2* is a target gene of E2F1, and that Ror2 suppresses Foxo3a-mediated expression of the cyclin-dependent kinase inhibitors p21 and p27 via the PI3K-Akt pathway, leading to activation of E2F1-mediated transcription, thereby promoting the G1/S-phase transition (Endo et al., 2020). Although Wnt5a is not required for the cell-cycle progression stimulated by bFGF, the possible involvement of other Wnt ligands, including Wnt5b and Wnt11, has to be investigated.

### Stem/Progenitor Cell Aging

It is known that the function of adult tissue stem/progenitor cells attenuates with aging that is causally linked to the age-associated impairment in tissue repair, regeneration, and homeostasis. Therefore, it is expected to develop clinical intervention methods for preventing tissue aging, by targeting tissue stem/progenitor cell aging. Dental pulp stem cells (DPSCs) play an important role in maintaining tooth homeostasis and repairing of postnatal tooth (Zheng et al., 2019). Furthermore, DPSCs exhibit multipotent differentiation capacity, and thus have the potential for use in clinical applications not only for dental diseases, but also for systemic diseases (Yamada et al., 2019). They can be isolated from human dental pulp tissues and expanded in culture, but gradually lose their proliferative ability and multipotent differentiation potential during the expansion because of entering cellular senescence. In cultured human DPSCs, *Ror2* is expressed highly at earlier passages, but its expression is decreased in senescent DPSCs (Dong et al., 2021). Consistently, decreased expression of *Ror2* is also detected in DPSCs isolated from elderly donors compared to those from young donors. Furthermore, Ror2 can inhibit induction of cellular senescence in cultured DPSCs by inhibiting STK4-Foxo1 pathway in a Wnt5a-independent manner (Dong et al., 2021). Therefore, supplementing expression of *Ror2* or inhibiting decreased expression of *Ror2* in DPSCs might lead to the maintenance of their stemness, which might be useful for the clinical use of DPSCs.

On the other hand, Wnt5a can induce cellular senescence in the tissue stem cells, including hematopoietic and hair follicle stem cells (Florian et al., 2013; Tiwari et al., 2021), although its receptors in regulating the cellular senescence remain elusive. In tendon stem/progenitor cells, however, Ror2 has been shown to mediate their Wnt5a-induced cellular senescence via JAK-STAT pathway (Chen et al., 2021). Therefore, it is important to clarify how Ror2 can regulate cellular senescence of these stem/progenitor cells in a cell-type specific manner.

## The Ror-Family Receptors in Age-Related Diseases

### Inflammatory Diseases

Recently, it has become evident that chronic inflammation is a pervasive feature of aged tissues, and is also the common cause of various age-related diseases, including fibrosis, arteriosclerosis, and neurodegenerative diseases. Therefore, it’s a provocative issue to uncover the molecular mechanisms underlying the development or progression of the age-related diseases due to chronic inflammation, aiming to develop novel clinical intervention methods to prevent or ameliorate the pathologies of these age-related diseases. In this regard, enhanced and/or sustained activation of Ror1/Ror2-mediated signaling induced by prolonged inflammation might be associated with progression of these age-related diseases. In the kidney, expression levels of *Ror1*, *Ror2*, and *Wnt5a* are increased in the progressive fibrotic tissues with persistent inflammation after injury (Li et al., 2013). In the damaged kidneys from *Ror2* ^+/−^ mice, reduced disruption of the tubular basement membrane (TBM) along with reduced expression of *MMP-2* in tubular epithelial cells were observed compared to *Ror2* ^+/+^ mice (Li et al., 2013), suggesting that Wnt5a-Ror2 signaling might play an important role in disrupting TBM via MMP-2 during renal fibrosis. Furthermore, Wnt5a and Ror2 are expressed highly in foam cells within the atherosclerotic plaque (Ackers et al., 2018; Zhang C.-J. et al., 2020). Aberrant expression of Wnt5a in vascular smooth muscle cells (VSMCs) reduces expression of adenosine triphosphate-binding cassette A1, a key cholesterol transporter, via Ror2, resulting in the enhancement of cholesterol accumulation and inflammatory response in VSMCs (Zhang C.-J. et al., 2020), suggesting that Wnt5a-Ror2 signaling plays a critical role in the pathogenesis of atherosclerosis. A critical role of Wnt5a-Ror2 signaling has also been reported in the dextran sodium sulfate-induced colitis mouse model, where sustained up-regulation of Wnt5a can be observed in stromal fibroblasts in the ulcerative lesions of these mice, and Wnt5a-Ror2 signaling activated in dendritic cells can promote interferon-γ signaling, thereby promoting colitis (Sato et al., 2015). *Ror2* expression is also increased in degenerating neurons by IL-1β secreted from activated microglia in the spinal cord of mice with experimental autoimmune encephalomyelitis, a mouse model of multiple sclerosis (Shimizu et al., 2016). Wnt5a- or Wnt11-Ror2 signaling can mediate IL-1β-induced neuronal cell death, suggesting that Ror2-mediated signaling might promote the pathology of the neurodegenerative diseases.

### Cancer Progression

Since most of cancers are developed in elder people than young people, it is conceivable that aging and cancer development are tightly related. Accumulating evidence demonstrates that age-related chronic inflammation plays important roles in the progression of cancers (Coussens and Werb, 2002; Ostan et al., 2015). Interestingly, Ror1/Ror2 are expressed highly in various types of cancers (Zheng et al., 2016; Bayerlová et al., 2017; Hossein et al., 2017; Hasan et al., 2018; Astudillo, 2021). Enhanced expression of Ror1 and/or Ror2 in cancer cells can promote their proliferation, migration, invasion, survival or chemoresistance through activation of Rho-family GTPases, c-Src, MAP kinases or Akt in Wnt5a-dependent and/or -independent manners (Enomoto et al., 2009; Zhang et al., 2012; Ida et al., 2016; Nishita et al., 2017; Hasan et al., 2019). In addition, several studies have indicated that Ror1 might be ideal therapeutic target of cancers including leukemia and breast cancer (Karvonen et al., 2017; Choi et al., 2018; Wallstabe et al., 2019; Zhang et al., 2019; Stüber et al., 2020). These studies surmise that increased expression of Ror1 and/or Ror2 in cancer cells is important for their progression. Here, we describe the mechanism how expression of Ror1/Ror2 is upregulated in cancer cells by taking aging into account (see below).

Accumulating evidence has shown that expression of the Ror-family receptors in cancer cells can be regulated by multiple factors and drugs (Table 1). In breast cancers, increased expression of *Ror1* can be attributable to reduced expression of *miR30a*, a suppressor of *Ror1* (Wang et al., 2018), which is up-regulated by aging, and age-related up-regulation of *miR30a* induces cellular senescence (Muther et al., 2017). *miR30a* can inhibit expression of *Ror1* to suppress the progression of breast cancer, however, its expression level is reduced in breast cancer cells, resulting in up-regulation of *Ror1* and promotion of the progression of breast cancer.

**TABLE 1:** Factors and drugs regulating expression of the Ror-family receptors in cancer cells.

Types of Cancers	Regulated Genes	Analyzed Samples (Cell line, Clinical Sample *etc.*)	Regulatory Factors	References
Breast cancer	Ror1	MDA-MB 231	Induced by activation of glucocorticoid receptor	Obradović et al. (2019)
	Ror1	HCC1954	Induced by YAP1	Islam et al. (2019)
	Ror1	Hs578T, MDA-MB 231	Induced by Twist	Cao et al. (2018)
	Ror1	BT549, MDA-MB 231	Inhibited by *miR30a*	Wang et al. (2018)
	Ror1	Patient derived xenograft	Induced by treatment with paclitaxel	Zhang et al. (2019)
Ovarian cancer	Ror1	Patient derived primary cell	Induced by dexamethasone	Karvonen et al. (2020)
		JHOS2, Ovsaho, Kuramochi		
	Ror2	Patient sample	Induced along with cisplatin resistance	Veskimäe et al. (2018)
		A2780		
	Ror2	SKOV3	Induced by STAT3	Arabzadeh et al. (2016)
	Ror1	SKOV3, COV434	Inhibited by *miR382*	Tan et al. (2016)
Gastric cancer	Ror1	MKN45	Induced by STAT3	Ikeda et al. (2020)
	Ror1	AGS, BGC823	Inhibited by *miR27b-3p*	Tao et al. (2015)
Leukemia	Ror1	Patient sample	Induced by STAT3	Li et al. (2010)
	Ror1	Patient sample	Inhibited by berberine	Mohammadlou et al. (2021)
	Ror1	RCH-ACV	Induced by UHRF1	Chow et al. (2018)
Lung cancer	Ror1	H1975, SK-LC-5	Induced by NKX2-1	Yamaguchi et al. (2012)
	Ror1	H1975, PC9, H441, H1299, H2228, HCC4006	Decreased by geldanamycin (Inhibitor of HSP90)	Khaledian et al. (2021)
	Ror1	Gefitinib resistant PC9, erlotinib resistant HCC827	Inhibited by miR30a-5p	Yang et al. (2021)
	Ror1	HCC827	Induced by STAT3	Wang et al. (2021)
Pancreatic cancer	Ror1	Panc1, Mia PaCa1	Induced by SETD8	Liu et al. (2021)
	Ror2	HPDE, HPDE/KRAS	Induced by conditioned medium obtained from adipocytes	Carbone et al. (2018)
Melanoma	Ror1	UACC1273	Inhibited by hypoxia	O'Connell et al. (2013)
	Ror2	UACC1273	Induced by hypoxia	
Head and neck squamous cell carcinoma	Ror2	UPCI: SCC152	Induced by E6/E7	Avincsal et al. (2021)
	Ror2	SNU899, TU177	Inhibited by *miR338-3p*	Yang et al. (2022)
Renal cancer	Ror2	786-0	Induced by HIF1α and HIF2α	Wright and Rathmell, (2010)

Furthermore, up-regulated expression of *Ror1* can be mediated by STAT3 in various types of cancer cells, thereby promoting proliferation of cancer cells in Wnt5a-dependent or -independent manners (Li et al., 2010; Ikeda et al., 2020; Wang et al., 2021). STAT3 has been shown to be activated in several aged tissues with chronic inflammation (Chazaud and Mouchiroud, 2014; O'Brown et al., 2015), and thus might contribute to the age-related progression of cancers through expression of *Ror1*. In the case of gastric cancers, constitutive Wnt5a-Ror2 signaling in bone marrow-derived mesenchymal stem cells (MSCs) induces expression of *CXCL16*, and that CXCL16 secreted from MSCs promotes proliferation and migration of undifferentiated gastric cancer cell line MKN45 cells through inducing expression of *Ror1* via STAT3 activation (Takiguchi et al., 2016; Ikeda et al., 2020). Considering that senescent MSCs secrete cytokines and chemokines (Lunyak et al., 2017), it can be assumed that aging might be one of critical factors promoting progression of cancers.

## Conclusion and Perspectives

**FIGURE 2:**
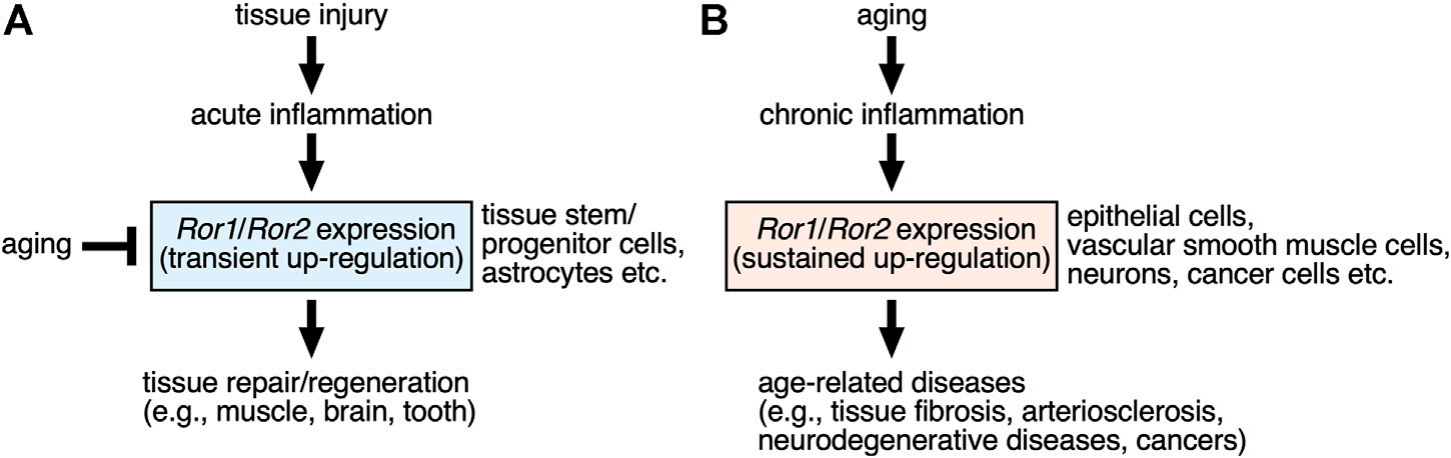
| Possible roles of the Ror-family receptors up-regulated by injury and aging in the tissue repair/regeneration and the age-related diseases. **(A)** Expression levels of Ror1 and/or Ror2 are increased transiently in somewhat restricted (specific) cells, including stem/progenitor cells and astrocytes, within the damaged tissues following injury, via environmental cues including inflammatory cytokines and growth factors. Up-regulated Ror1/Ror2 in turn contribute to the promotion of tissue repair or regeneration. Aging can mediate decreased expression of the Ror-family receptors in the tissue stem/progenitor cells, resulting in the dysfunction of these tissue stem/progenitor cells in regulating tissue repair or regeneration. **(B)** Sustained expression of Ror1 and/or Ror2 are induced in various types of cells under chronic inflammation caused by aging, thereby contributing to the development or progression of the age-related diseases, including fibrosis, arteriosclerosis, neurodegenerative diseases, and various cancers.

The Ror-family receptors play important roles in establishing developmental morphogenesis and tissue-/organo-genesis in redundant, yet pleiotropic manners. The activation of the Ror1/Ror2-signaling might be regulated by the induction of their expression by themselves, in addition to the stimulation with their ligands, including Wnt5a. Although expression levels of *Ror1*/*Ror2* are kept lower in most of adult tissues than developing ones, expression of *Ror1* and/or *Ror2* are increased transiently in specific cells, including stem/progenitor cells, following injury or inflammation, via environmental cues including inflammatory cytokines, contributing to the promotion of tissue repair or regeneration (Figure 2A). Furthermore, the Ror-family receptors seem to play a role in maintaining the stemness of tissue stem/progenitor cells, and thereby preventing the induction of their cellular senescence. Therefore, it can be assumed that decreased expression of the Ror-family receptors in the tissue stem/progenitor cells might lead to the dysfunction of these tissue stem/progenitor cells.

On the other hand, expression of *Ror1* and/or *Ror2* might be induced in various types of cells under chronic inflammation associated with aging, thereby contributing to the development or progression of the age-related diseases, including neurodegenerative diseases and cancers (Figure 2B). Therefore, it will be interesting to clarify the molecular mechanisms how expression of Ror1/Ror2 can be regulated in association with aging or cellular senescence.
